# Genome editing of the HIV co-receptors CCR5 and CXCR4 by CRISPR-Cas9 protects CD4^+^ T cells from HIV-1 infection

**DOI:** 10.1186/s13578-017-0174-2

**Published:** 2017-09-09

**Authors:** Zhepeng Liu, Shuliang Chen, Xu Jin, Qiankun Wang, Kongxiang Yang, Chenlin Li, Qiaoqiao Xiao, Panpan Hou, Shuai Liu, Shaoshuai Wu, Wei Hou, Yong Xiong, Chunyan Kong, Xixian Zhao, Li Wu, Chunmei Li, Guihong Sun, Deyin Guo

**Affiliations:** 1 School of Basic Medical Sciences, Wuhan University, Wuhan, 430072 People’s Republic of China; 2 Center for Retrovirus Research, Department of Veterinary Biosciences, The Ohio State University, Columbus, USA; 3 Guangxi Center for Disease Control and Prevention, Nanning, Guangxi People’s Republic of China; 4 College of Life Science, Wuhan University, Wuhan, People’s Republic of China; 5 Zhongnan Hospital, Wuhan University, Wuhan, People’s Republic of China; 6 School of Medicine (Shenzhen), Sun Yat-sen University, Guangzhou, 510080 People’s Republic of China

**Keywords:** CRISPR-Cas9, CCR5 and CXCR4 simultaneous, HIV-1, AIDS

## Abstract

**Background:**

The main approach to treat HIV-1 infection is combination antiretroviral therapy (cART). Although cART is effective in reducing HIV-1 viral load and controlling disease progression, it has many side effects, and is expensive for HIV-1 infected patients who must remain on lifetime treatment. HIV-1 gene therapy has drawn much attention as studies of genome editing tools have progressed. For example, zinc finger nucleases (ZFN), transcription activator like effector nucleases (TALEN) and clustered regularly interspaced short palindromic repeats (CRISPR)-Cas9 have been utilized to successfully disrupt the HIV-1 co-receptors CCR5 or CXCR4, thereby restricting HIV-1 infection. However, the effects of simultaneous genome editing of CXCR4 and CCR5 by CRISPR-Cas9 in blocking HIV-1 infection in primary CD4^+^ T cells has been rarely reported. Furthermore, combination of different target sites of CXCR4 and CCR5 for disruption also need investigation.

**Results:**

In this report, we designed two different gRNA combinations targeting both CXCR4 and CCR5, in a single vector. The CRISPR-sgRNAs-Cas9 could successfully induce editing of CXCR4 and CCR5 genes in various cell lines and primary CD4^+^ T cells. Using HIV-1 challenge assays, we demonstrated that CXCR4-tropic or CCR5-tropic HIV-1 infections were significantly reduced in *CXCR4*- and *CCR5*-modified cells, and the modified cells exhibited a selective advantage over unmodified cells during HIV-1 infection. The off-target analysis showed that no non-specific editing was identified in all predicted sites. In addition, apoptosis assays indicated that simultaneous disruption of CXCR4 and CCR5 in primary CD4^+^ T cells by CRISPR-Cas9 had no obvious cytotoxic effects on cell viability.

**Conclusions:**

Our results suggest that simultaneous genome editing of CXCR4 and CCR5 by CRISPR-Cas9 can potentially provide an effective and safe strategy towards a functional cure for HIV-1 infection.

**Electronic supplementary material:**

The online version of this article (doi:10.1186/s13578-017-0174-2) contains supplementary material, which is available to authorized users.

## Background

Acquired immune deficiency syndrome (AIDS), caused by infection with human immunodeficiency virus type 1 (HIV-1), has threatened the health of individuals since it was first reported in the early 1980s [1, 2]. HIV-1 mainly infects and destroys primary CD4^+^ T cells, which can lead to opportunistic infections or other infectious diseases, and certain cancers [3, 4]. HIV-1 infection of CD4^+^ T cells involves binding of the viral protein gp120 to the primary cellular receptor CD4 and either of the co-receptors, CCR5 or CXCR4. Through binding to receptors, HIV-1 enters the cell by membrane fusion. Next, the viral genomic RNA is converted into double-stranded DNA by HIV-1 reverse transcriptase [5, 6]. The viral DNA integrates into the host cell genome and is transcribed into a single 9 kb viral transcript. During viral replication, the integrated provirus can be transcribed into viral RNAs, which function as new copies of the virus genome and are packaged into new virus particles [7–9]. The most common antiretroviral therapy (ART) is based on the inhibition of multiple viral proteins which are involved in various processes of the HIV life cycle [10]. However, limitations of ART, such as high cost, long-term treatment, and chronic hepatic or cardiovascular system injury, inevitably necessitates the development of alternative and more effective therapies against HIV-1 infection [11–14].

In 1996, findings were reported that some Europeans who experienced a high-risk infectious event continued to live free of HIV-1 infection. These infection-free outcomes were found to be due to a natural 32 base-pair deletion in the CCR5 locus of these individuals [15, 16]. Further studies confirmed that people who lack a functional CCR5 co-receptor, due to the mutation, were resistant to HIV-1 infection [17]. In 2007, an American patient with HIV-1 infection and acute myeloid leukemia (AML) obtained a bone-marrow transplant from a CCR5-delta 32 donor for leukemia therapy, which also cured his HIV-1 infection without further ART [18, 19]. Based on these findings, the question has been proposed whether CCR5 or CXCR4 knockout, either singly or simultaneously, can confer HIV-1 resistance in patients. Scientists have addressed this question using different methods, from utilizing zinc finger nucleases (ZFN) to recent studies with CRISPR-Cas9 technology [20–23]. For example, Perez et al. generated an endogenous CCR5 disrupted genotype using ZFN, and achieved permanent CCR5 disruption in 50% of the primary CD4^+^ T cell population, which showed significant resistance to HIV-1 infection in vivo and in vitro [24].

Clustered regularly interspaced short palindromic repeats (CRISPR) and CRISPR associated nuclease 9 (CRISPR-Cas9) gene modification technique was first developed in 2013, which has resulted in a revolution of gene modification [25–27]. The CRISPR/Cas9 system was initially identified as an adaptive immune system in bacteria and archaea [28], which mainly consist of a non-specific endonuclease Cas9 and a sequence-specific sgRNA. After the guide RNA is transcribed to pre-crRNA, it will be processed by RNAase III or another nuclease into a mature transcript. When it binds to a target sequence, it will lead to a double-strand break (DSB) in the target DNA sequence by Cas9 cleavage, and this will be repaired by homologous directed repair or non-homologous end joining (NHEJ) [26, 27, 29–31]. According to a previous study, NHEJ plays a major role and leads to nucleotide mutation, insertion, and frame shift [32]. As the CRISPR/Cas9 technology has emerged, it was immediately utilized to treat HIV-1 infection. Wang et al. designed sgRNAs to disrupt *CCR5* using a lentiviral system expressing Cas9 and the sgRNA. They utilized this system to generate CD4^+^ T cells that showed high frequencies of CCR5 disruption with no mismatch in all predicted off-target sites [33]. In most cases of HIV-1 infection, although HIV-1 uses CCR5 to mediate entry to cells, CXCR4 can function as a co-receptor at the late stages of infection, which contributes to disease progression [34–36]. Our group also reported that disruption of the CXCR4 co-receptor by CRISPR-Cas9 resulted in protection of primary CD4^+^ T cells from HIV-1 infection [37]. However, to date, only one study has investigated simultaneous CXCR4 and CCR5 modification using CRISPR-Cas9, which was reported to inhibit HIV-1 infection in cells [38]. In this study only one combination of CXCR4 and CCR5 sgRNA was assessed. For efficacy and safety concerns, multiple combinations of sgRNAs of CXCR4 and CCR5 should be assessed.

In our previous study, the two targeting CXCR4 sgRNAs and Cas9 efficiently inhibited HIV-1 infection in CD4^+^ T cells [37]. Here, we report that each of the two CXCR4 sgRNA together with one CCR5 sgRNA, combined in one vector (lenti-X4R5-Cas9-#1, lenti-X4R5-Cas9-#2), can disrupt CXCR4 and CCR5 simultaneously in various cell lines, as well as primary CD4^+^ T cells. Importantly, the modified cells are resistant to CXCR4-tropic or/and CCR5-tropic HIV-1 infection and exhibit a selective advantage over unmodified cells throughout the HIV-1 infection period. We further verified that the lenti-X4R5-Cas9 could work safely without any non-specific editing or cytotoxicity after CXCR4 and CCR5 disruption. Therefore, this study provides a basis for the potential use of the CRISPR-Cas9 system to efficiently block HIV-1 infection in patients.

## Methods

### Lenti-X4R5-Cas9 construct

**Fig. 1:**
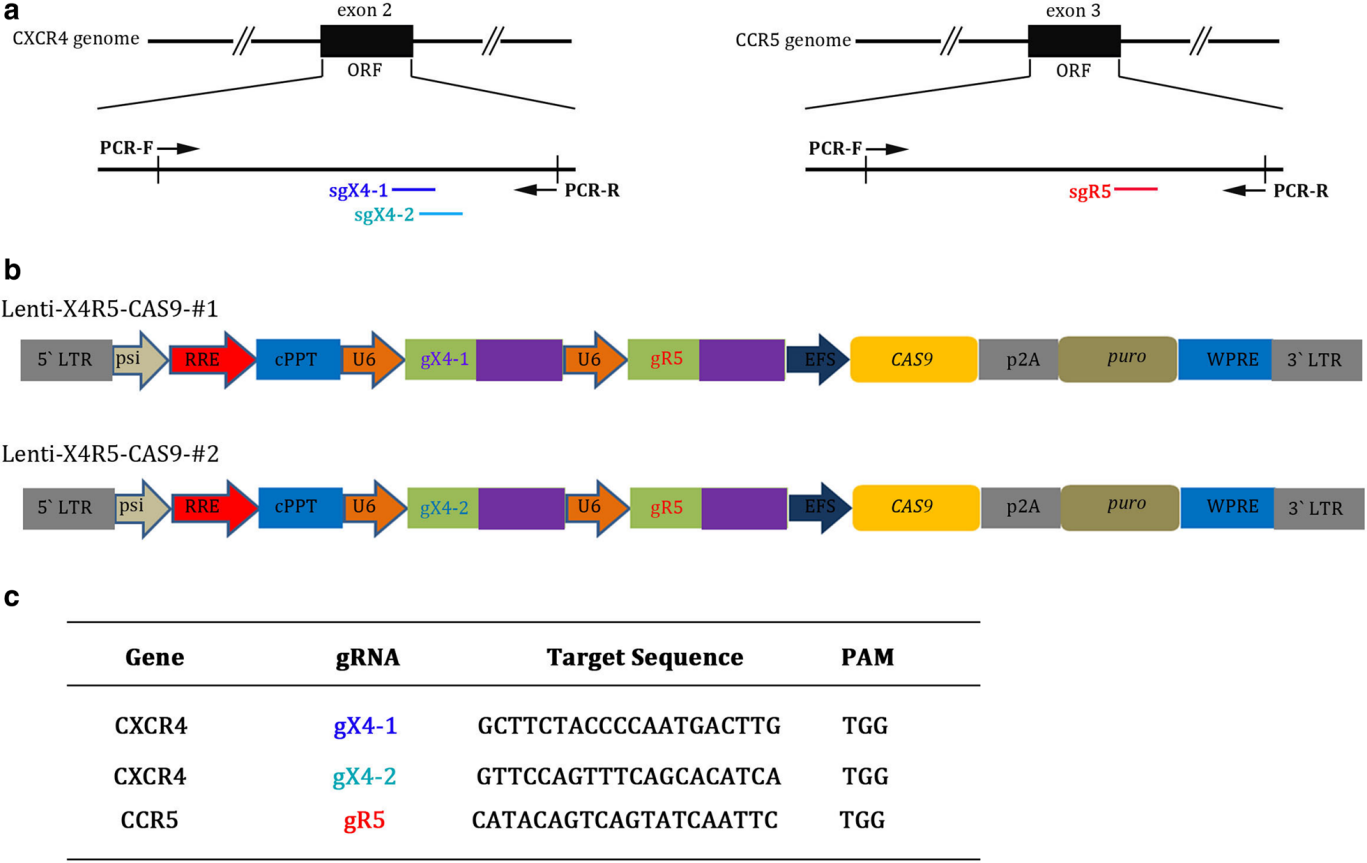
Schematic diagram of sgRNA of CXCR4 and CCR5 targets and vector construction. **a** Schematic of the CXCR4 and CCR5 coding region in genomic DNA sequences targeted by lenti-X4R5-Cas9-#1,#2. **b** Structure of lenti-X4R5-Cas9-#1,#2 vectors expressing Cas9 and dual sgRNA. **c** gRNA sequences used in lenti-X4R5-Cas9-#1,#2 vectors

The sgRNA for CXCR4 or CCR5 were designed and synthesized as previously described [37, 39]. To generate constructs to target both CXCR4 and CCR5, the lenti-sgR5-Cas9 vector, containing the gRNA targeting CCR5 region, was inserted by the different CXCR4 targeting sgRNAs containing crRNA-loop-tracrRNA. Briefly, U6-gX4-1/-2-crRNA-loop-tracrRNA was amplified and inserted into lenti-sgR5-Cas9 vector digested with Pac1 and Kpn1. The corresponding primers and gRNAs were listed in Additional file 1: Table S1 and Fig. 1.

### Cell lines culture and primary CD4^+^ T cell isolation

TZM-bl cells, Jurkat T cells and human CD4^+^ T cells were cultured and prepared as previously described [37]. The human blood samples for primary CD4^+^ T isolation were taken from healthy donors in Wuhan Blood Center (Wuhan, China), and the peripheral blood mononuclear cells (PBMC) were isolated with lymphocyte separation medium Ficoll-paque Premium (BD). The primary CD4^+^ T cells in PBMC were separated and enriched using a human CD4^+^ T cell isolation kit (Miltenyi Biotech), according to the manufacturer’s instructions. Primary CD4^+^ T cells were cultured in RPMI 1640 medium and stimulated by CD3/CD28 in the presence of recombinant human interleukin-2 (IL-2, 10 IU/ml) for 3 days.

### Lentivirus and HIV virus (HIV-1_NL4-3_ and HIV-1_YU-2_) production and transduction

X4R5-Cas9 lentivirus or control lentivirus were produced as previously described [37]. Briefly, 6.0 µg lenti-X4R5-Cas9 or control vector, 4.5 µg psPAX2 and 3.0 µg pMD2.G were co-transfected into 293T cells seeded in a 10 cm plate using Polyethylenimine (PEI) transfection reagent. The supernatant was collected and filtered with 0.45 µm filter. The virus was stored at −80 °C. The spin-transduction of Jurkat T cells was performed at 1200 g for 2 h and 8 µg/ml polybrene (Sigma) was used to facilitate the transduction efficacy. The supernatant of the lentivirus transduced Jurkat T cells were then replaced with RPMI containing 10% FBS for further culture and study. The production of HIV-1 strains, CXCR4-tropic HIV-1_NL4-3_ and CCR5-tropic HIV-1_YU-2_ were performed as previously reported [40].

### Primary CD4^+^ T cell nucleofection

The transfection of primary CD4^+^ T cells was performed as previously described [37]. In short, 5x10^6^ primary CD4^+^ T cells were centrifuged and washed with 1xPBS twice, and the cell were re-suspended with 100 µl prepared nucleofector buffer and 2 µg corresponding X4R5-Cas9 or control plasmids. The mixture was then transferred to the specific cuvette and electro-transfected with primary cell associated program in a Lonza 4D-Nucleofector System. After transfection, the cells were transferred to a CD3/CD28 coated six well plate and cultured with RPMI 1640 supplemented with 10% FBS, and IL-2 (20 IU/ml).

### HIV-1 infection

CXCR4 and CCR5-modified or control cells (2.5 × 10^5^) were collected and washed three times, and then CXCR4-tropic HIV-1_NL4-3_ or/and CCR5-tropic HIV-1_YU-2_ was added to the cell culture medium at MOI = 0.1 and incubated for 8 h. After the incubation, the infected cells were then collected and washed three times with 1×PBS, and the cells were replenished with RPMI1640. The viral load of the supernatant was then detected by p24 ELISA at indicated days post-infection according to our previous report [37].

### T7 endonuclease I assay (T7E1)

Genomic DNA was extracted from modified or control cells using Blood and Cell culture DNA Midi Kit (Tiangen, China). After PCR amplification of CXCR4 or CCR5 fragment, T7 endonuclease 1 assay was performed according to manufacturer’s instruction with 400 ng PCR product annealed and digested with T7 endonuclease 1 (10 units/µl, NEB) and analyzed with ethidium bromide (EB) stained 1.5% agarose gel. For on-target analysis of sgRNA, the fragments were ligated with pGEM-T easy vector (Promega), and then analyzed by DNA sequencing or deep sequencing.

### Selective advantage analysis of genome-disrupted cells after HIV-1 challenge

To test whether CXCR4 and CCR5 disrupted cells have a selection advantage over un-edited cells during HIV-1 infection, a selective advantage assay was performed as previously described [20, 24, 33]. The Jurkat T cells were transduced with X4R5-Cas9-#1,#2, or control associated lentivirus at MOI = 10. After 4 days, the transduced cells or mock-transduced cells were challenged with mixture of HIV-1_NL4-3_ and HIV-1_YU-2_ (1:1) at MOI = 0.1 for 8 h. Cells were then washed by 1xPBS three times and cultured in fresh medium. After HIV-1 challenge, the cells and supernatants were collected every 3 days and replenished with fresh cell culture medium for a total of 18 days. The genomic DNA of cells at day 0, 9, and 18 post-infection were extracted and subjected to T7E1 assay.

### Flow cytometry analysis of CXCR4 and CCR5 expression levels

To detect CXCR4 and CCR5 expression on the cell surface after 3 days after transfecting lenti-X4R5-Cas9 into cells and bearing with puromycin (1 µg/ml) selection for 24 h, Flow cytometry was used to determine the relative knockout efficacy. Briefly, 2.5 × 10^5^ modified or control cells were collected and washed three times in 1xPBS, and then the cells were stained with PE-conjugated anti-CXCR4 and APC-conjugated anti-CCR5 (Biolegend) for 15 min at 4 °C. The cells were then washed for three times after incubation. The relative expression levels of CXCR4 and CCR5 were then analyzed by flow cytometry (Aria1ll, BD).

### Off-target site analysis

To predict the site specificity of gRNAs of CXCR4 and CCR5 in genome, mismatches of designed sequences were predicted with the online tool (http://crispr.mit.edu). The off-target sites (2 for gX4-1, 5 for gX4-2 and 8 for gR5) were amplified from genomic DNA of edited cells and analyzed with T7E1 or sequencing.

### Deep sequencing

Target loci were amplified by the specific primer as shown in Additional file 1: Table S1. Before sequencing on an Illumina HiSeq 2500 platform, the amplicons were purified, end-repaired and connected with sequencing primer. For the sequences gained by sequencing, low-quality and joint pollution data were removed to obtain reliable target sequences (clean reads) for subsequent analysis. The corresponding Read1 and Read2 (sequences gained from the 5′ and 3′ ends, respectively) were spliced. ClustalX2 software was then used for sequence alignment.

### Primary CD4^+^ T cell apoptosis analysis

Primary CD4^+^ T cells (5 × 10^6^) were electro-transfected with 2 µg lenti-X4R5-Cas9 or control plasmid as described previously [37]. To assess the effect of both CXCR4 and CCR5 editing on cell apoptosis, the Annexin V Apoptosis detection kit 1 (BD Pharmingin) was used to evaluate early apoptosis. The treated cells were then analyzed with flow cytometry. The data were analyzed by FlowJ.

### Data analysis and statistics

All data are presented as the mean ± SD (standard deviation). Statistical analyses were performed using SPSS software, version 16.0 (SPSS, Chicago, IL, USA). Differences between two groups were analyzed using the Unpaired Student’s *t* test, and difference between multiple groups were evaluated using one-way analysis of variance. Differences with *p* < 0.05 were considered statistically significant.

## Results

### Lenti-X4R5-Cas9 mediated CXCR4 and CCR5 disruption protects TZM-bl cells from HIV-1 infection

To disrupt the HIV co-receptor of CXCR4 and CCR5 concurrently at genome level, we amplified and inserted our previously reported sgRNAs of CXCR4 with U6 promoter into lenti-sgR5-Cas9 [37]. The schematic diagram of sgRNA selection and a sketch of vector construction were presented (Fig. 1a, b). Meanwhile, the specific sequences of sgRNAs used were listed (Fig. 1c), The two constructed plasmids were referred to as lenti-X4R5-Cas9-#1(or#1) and lenti-X4R5-Cas9-#2 (or#2).

**Fig. 2:**
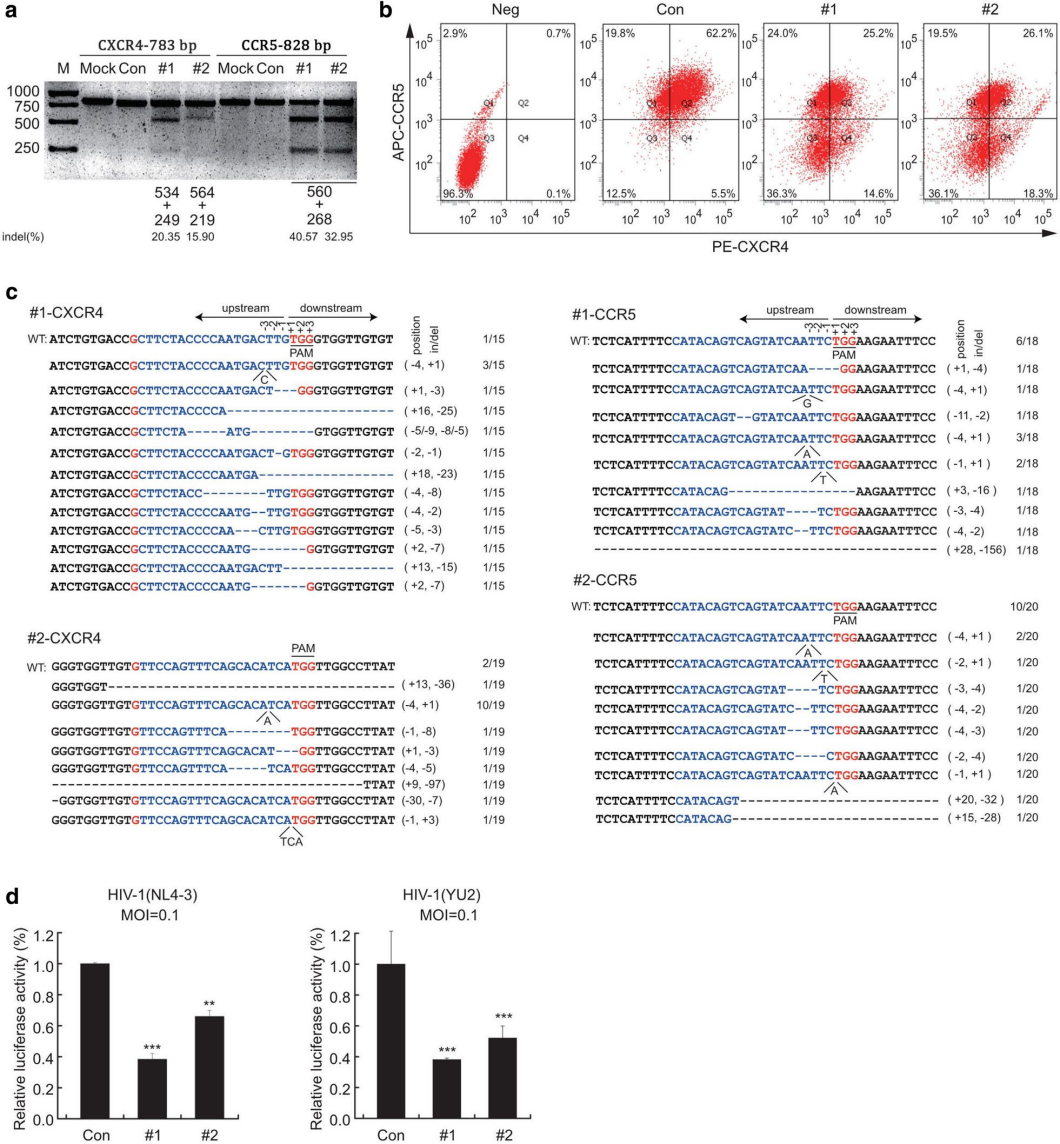
Disruption of CXCR4 and CCR5 protects TZM-bl cells from HIV-1 infection. **a** T7E1 assay for genome level cleavage efficacy by lenti-X4R5-Cas9-#1,#2 in TZM-bl. **b** Expression of CXCR4 or CCR5 in TZM-bl cell line transfected with lenti-X4R5-Cas9 by lipo2000 transfection reagent were analyzed with flow cytometry. **c** On-target analysis of the cleavage on target sites. **d** lenti-X4R5-Cas9 transfected TZM-bl cell line challenged with HIV-1_NL4-3_ or HIV-1_YU-2_ (3 days post infection). The data shown were the mean ± SD of three independent experiments. *P < 0.05; **P < 0.01; ***P < 0.001; *t* test

To test the efficacy of lenti-X4R5-Cas9 directed simultaneous disruption of CXCR4 or CCR5 in cells, the epithelial origin TZM-bl cell line was selected first, for the reason that it was widely used as CD4^+^ cells in HIV-1 neutralization assay with expression of co-receptor CXCR4 and CCR5 on cell surface. Since the plasmids we constructed contain the selection marker puromycin but not EGFP, the EGFP expressing plasmid was used as indicator to make sure that the lenti-X4R5-Cas9 were transfected into the cells successfully. The cells were treated with 1 µg/ml puromycin for 24 h to kill the unmodified cells and improved the transfection efficiency. We performed the T7E1 assay to measure the insertion/deletion (indel) efficacy at each target site of CXCR4 and CCR5. The results revealed that the indel mutation rate of 1#CXCR4, 2#CXCR4, 1#CCR5 and 2#CCR5 are 20.35, 40.57, 15.90 and 32.95%, respectively (Fig. 2a). The corresponding primers are listed in Additional file 1: Table S1. To measure changes of expression of CXCR4 and CCR5 on cell surface after lenti-X4R5-Cas9-#1 and lenti-X4R5-Cas9-#2 editing, flow cytometry was performed to directly evaluate CXCR4 and CCR5 expression levels 72 h post-transfection. We observed that simultaneous CXCR4 and CCR5 editing by lenti-X4R5-Cas9-#1 and lenti-X4R5-Cas9-#2, resulted in knockout efficacy of 23.8 and 23.6%, respectively, as compared to positive control (Fig. 2b). DNA sequencing confirmed the efficacy of on-target for each sgRNAs and obvious indels (insert/deletion) were detected in each target (Fig. 2c).

We next examined whether lenti-X4R5-Cas9-#1 and lenti-X4R5-Cas9-#2 mediated CXCR4 and CCR5 disruption could protect TZM-bl cells from HIV-1 infection. HIV-1_NL4-3_ strain (X4-tropic) and HIV-1_YU-2_ strain (R5-tropic) were used to infect TZM-bl cells and the HIV-1 levels were measured by luciferase reporter assay at 72 h post infection. The results indicated that both lenti-X4R5-Cas9-#1 and lenti-X4R5-Cas9-#2 mediated CXCR4 and CCR5 knockout conferred TZM-bl cells resistant to X4- or R5-tropic HIV-1 infections (Fig. 2d).

### Transduction of X4R5-Cas9 lentivirus protects Jurkat T cells from HIV-1 infection and the modified cells gain a selective advantage over unmodified cells

**Fig. 3:**
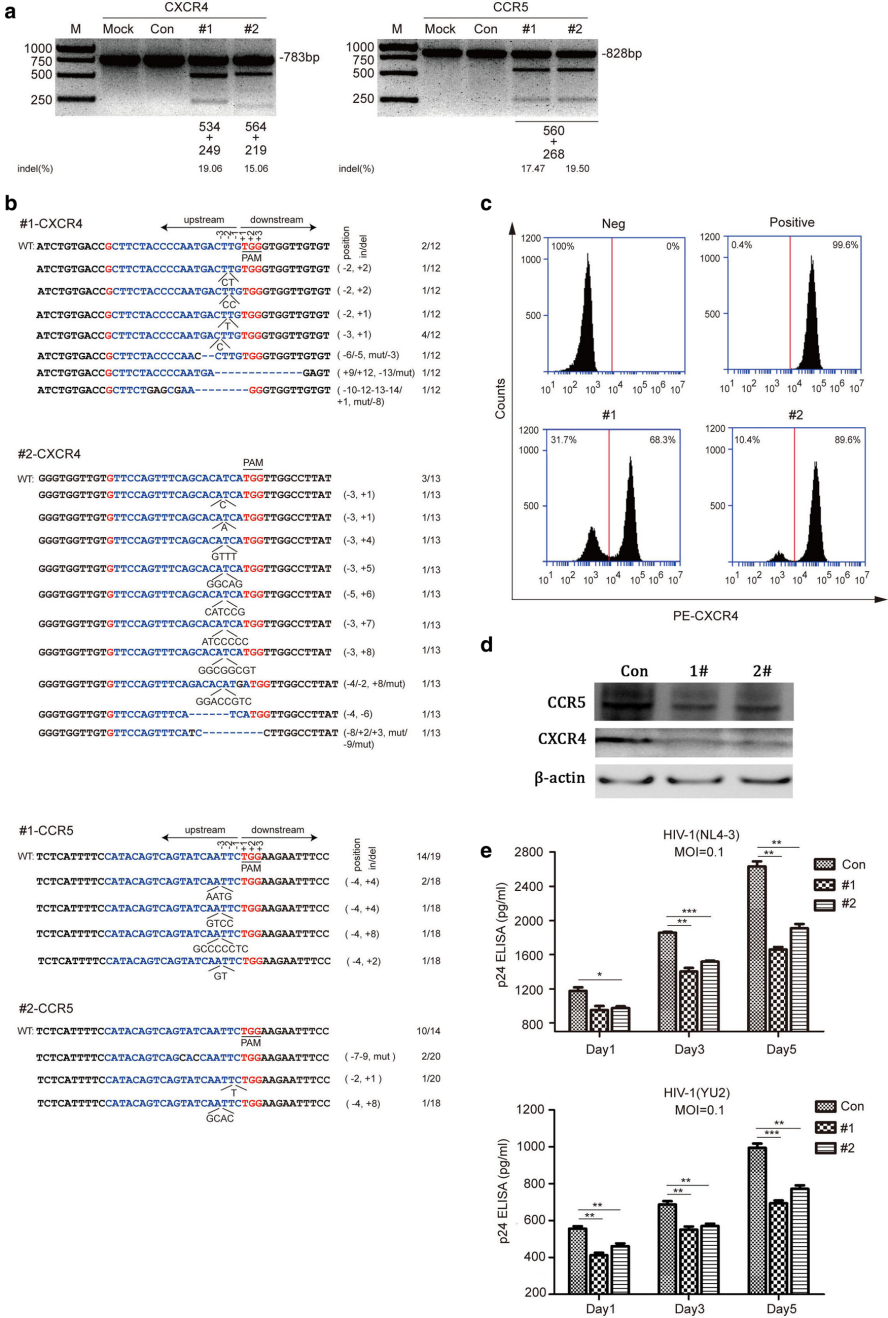
Jurkat T cell line modified by X4R5-Cas9 lentivirus antagonized HIV infection. **a** T7E1 assay identification of packaged X4R5-Cas9 lentivirus mediated cleavage at the genome level. **b** On-target analysis of each target in Jurkat T cells. **c** Flow cytometry analysis of CXCR4 expression on the cell surface. Jurkat T cell line was transduced with X4R5-Cas9 lentivirus at MOI = 40. CCR5 surface expression detection was excluded because of its low expression on Jurkat T cells. **d** Detection of protein level of CXCR4 and CCR5 after Jurkat cells were transduced with X4R5-Cas9 lentivirus. **e** HIV-1 titer change detected by p24 gag ELISA from day 1 to day 5 post-infection. The data shown were the mean ± SD of three independent experiments. *P < 0.05; **P < 0.01; ***P < 0.001; *t* test

The TZM-bl cells are of epithelial origin, and the acute T cell leukemia (ATCL) derived Jurkat T cells, like primary human CD4^+^ T cells, are suspension cells with expression of co-receptor CXCR4 and CCR5 on cell surface, which can be a perfect option for HIV-1 infection study. We further tested whether the two X4R5-Cas9 plasmids could work in Jurkat T cell line. Jurkat T cells were then transduced with X4R5-Cas9-#1 or X4R5-Cas9-#2 lentivirus and bearing with puromycin for selection. To evaluate X4R5-Cas9 lentivirus mediated knockout efficacy at the genome level, CXCR4 and CCR5 targets containing fragments were amplified with PCR, and analyzed with the T7E1 assay. The results showed that, at the genome level, both CXCR4 and CCR5 were disrupted in the lenti-X4R5- Cas9#1 or lenti-X4R5-Cas9-#2 edited Jurkat T cells (Fig. 3a). The analysis of on-target by DNA sequencing also verified that the targets had evident indels (insert/deletion) or mutations (Fig. 3b).

To observe the changes in protein expression level on cell surface, the treated cells were analyzed by flow cytometry. Because of low expression of CCR5 on the cell surface of Jurkat T cells, the analysis was performed with CXCR4 only. However, we detected the changes in total protein levels of both CXCR4 and CCR5 by western blot. The results of flow cytometry and western blot indicated that the expression level of either CXCR4 on cell surface, or total levels of CXCR4 and CCR5 were significantly down-regulated in Jurkat T cells (Fig. 3c, d).

We next examined whether simultaneous knockout of CXCR4 and CCR5 in Jurkat T cells rendered the modified cells resistant to HIV-1 infection. The HIV-1 challenge assay was performed with HIV-1_NL4-3_ or HIV-1_YU-2_, and the released p24 in supernatants were quantified by ELISA at indicated times post-infection. The p24 ELISA results showed that, compared with unmodified cells, the X4R5-gene modified cells produced less p24, indicating these cells were resistant to either CXCR4-tropic HIV-1_NL4-3_ or CCR5-tropic HIV-1_YU-2_ infection (Fig. 3e).

**Fig. 4:**
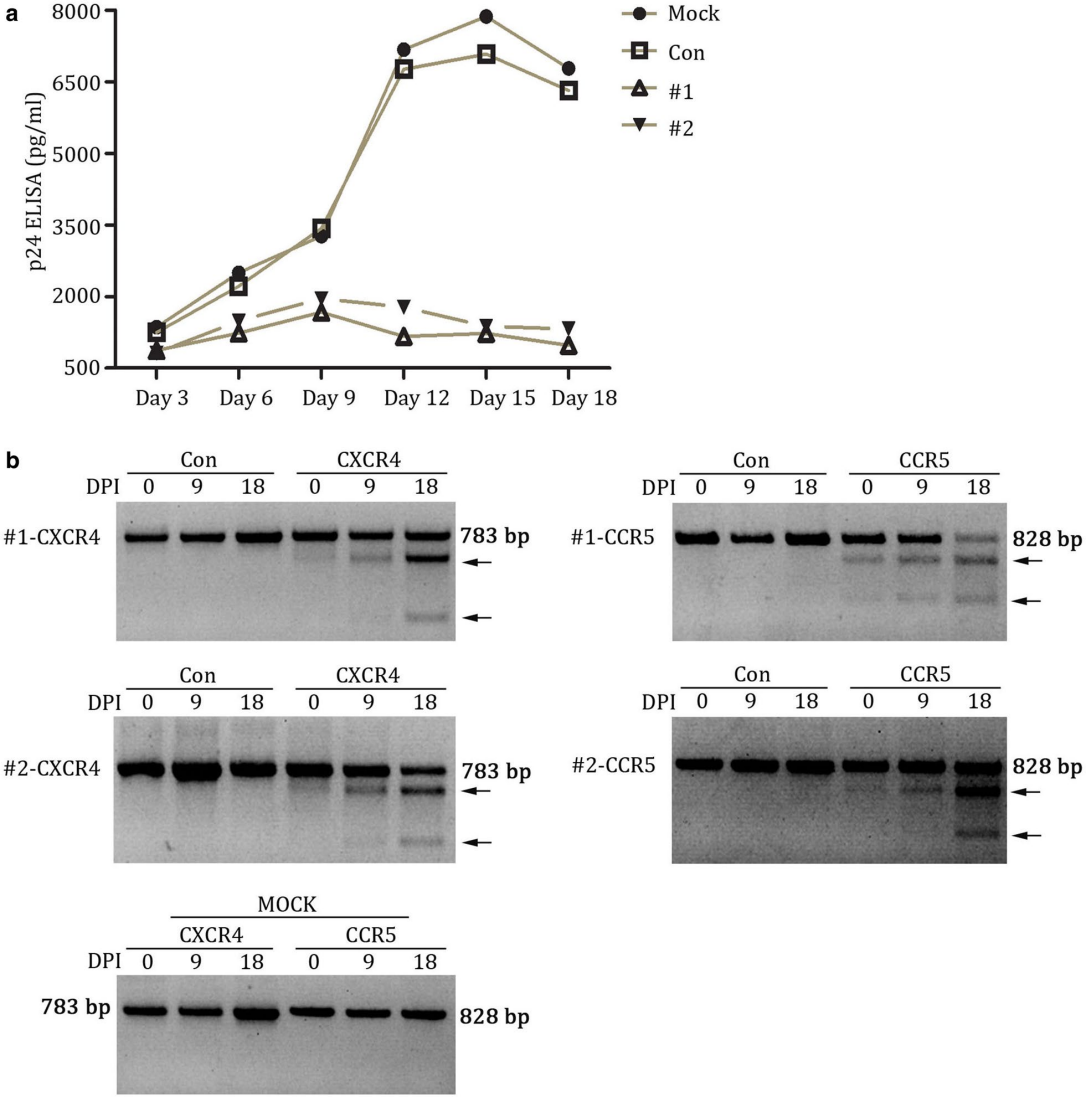
X4R5-Cas9 lentivirus modified Jurkat T cells were enriched after CXCR4-tropic (NL4-3) and CCR5-tropic (YU-2) HIV-1 challenge. **a** HIV replication in X4R5-Cas9 lentivirus modified as well as mock and control Jurkat T cells infected with X4-tropic and R5-tropic HIV-1 concurrently. Values represent the mean of duplicate infections. **b** cleavage analysis of CXCR4 and CCR5 by T7 endonuclease 1 in mock, control, lenti-X4R5-Cas9-#1 and lenti-X4R5-Cas9-#2 group at 0, 9 and 18 days after HIV-1 challenge. The lower migrating bands (indicated by *arrows*) in* each lane* indicate the disrupted CXCR4 and CCR5 alleles. *DPI* days post infection

In order to rule out the effect of multiple rounds of infection in unmodified cells, we performed the selective advantage assay by exposing the treated cells to both HIV-1_NL4-3_ and HIV-1_YU-2_ (1:1) simultaneously and prolonging the culture time for a total of 18 days. As measured by HIV-1 gag p24 in the culture supernatants, the cells of modified groups (#1 and#2) presented a slight rise in HIV-1 replication as compared with mock or control groups throughout the whole culture period (Fig. 4a). T7 endonuclease 1 analysis of amplicons of CXCR4 and CCR5 was performed using DNA extracted from all four groups at 0, 9, 18 days post-infection, and the result showed that the modified groups (#1 and #2) exhibited an obvious increase of lower migrating bands corresponding to cleavage products, but the unmodified groups (mock, control) had no cleavage products (Fig. 4b). These results suggest that the modified cells (#1 and #2) were enriched during the HIV-1 mix infection, and the cells were rendered with a relative step by step HIV-1 resistance increase. Thus, we concluded that the X4R5-gene modified Jurkat T cells are resistant to both HIV-1_NL4-3_ and HIV-1_YU-2_ virus and exhibit a selective advantage over unmodified cells during mixed HIV-1 infection.

### CXCR4 and CCR5-modified primary CD4^+^ T cells are protected from HIV-1 infection

**Fig. 5:**
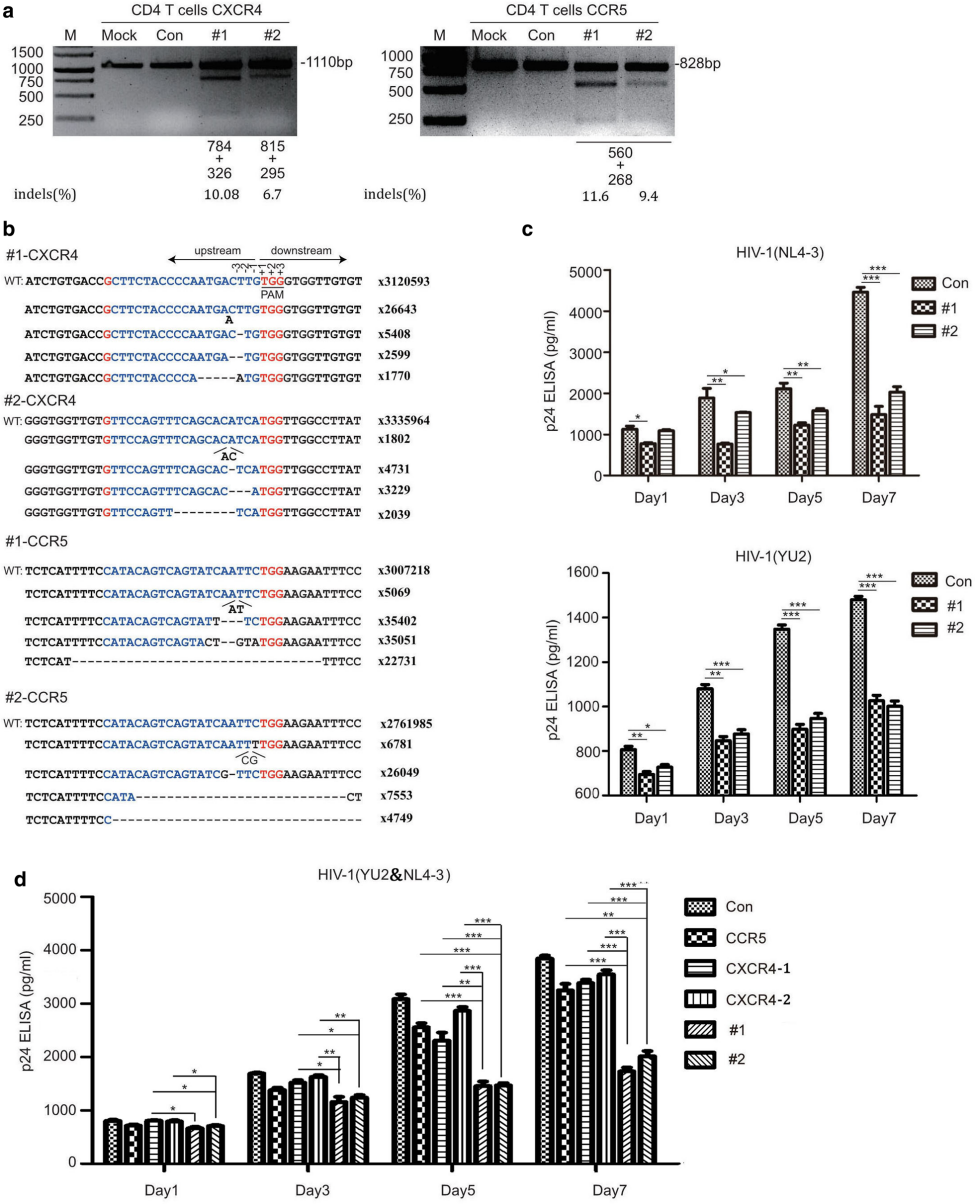
lenti-X4R5-Cas9 modified primary CD4^+^ T cell resists HIV challenge. **a** T7E1 analysis of CXCR4 and CCR5 disruption. **b** Deep sequencing analysis of typical NHEJ (indels) of related targets. **c** lenti-X4R5-Cas9 modified CD4^+^ T cell challenged with HIV-1_NL4-3_ or HIV-1_YU-2_. **d** lenti-X4R5-Cas9 modified CD4^+^ T cell exposed to dual-tropic HIV-1 variants (NL4-3 & YU-2, 1:1). The CCR5, CXCR4-1, CXCR4-2 represent single disruption of CCR5 or CXCR4, which use the same corresponding gRNAs used in lenti-X4R5-Cas9-#1 or #2. The data shown were the mean ± SD of three independent experiments. *P < 0.05; **P < 0.01; ***P < 0.001; *t* test

Human primary CD4^+^ T cells are the major target for HIV-1 infection. We next tested whether the lenti-X4R5-Cas9 system worked in primary CD4^+^ T cells. Like the treatment of Jurkat T cells, the packaged X4R5-Cas9 lentivirus was used to transduce the primary CD4^+^ T cells, however, the efficiency is very low as described in data very recent report [41]. We then turn to use electroporation to transfect the primary CD4^+^ T cells as described in our previously reported work [37]. After the plasmids transfection into human primary CD4^+^ T cells, we first analyzed the knockout efficacy at the genome level, and found that both CXCR4 and CCR5 genes were specifically edited as expected (Fig. 5a). DNA deep sequencing analysis of CXCR4 or CCR5 on-target efficacy showed that lenti-X4R5-Cas9 had efficiently disrupted either CXCR4 or CCR5 targets (Fig. 5b). We then tested whether the disruption of CXCR4 and CCR5 on primary CD4^+^ T cells protected cells from HIV-1 infection. HIV-1 p24 levels in the supernatants of infected cells were measured at 1, 3, 5 and 7 days post-infection and the results indicated that lenti-X4R5-Cas9 modified primary CD4^+^ T cells were protected from infection by either X4- or R5-tropic HIV-1 strains, compared to unmodified control (Fig. 5c). Furthermore, to explore the effect of disruption of both co-receptors on the protection against a dual-tropic HIV-1 variant instead of a distinct co-receptor tropism virus, we performed the HIV-1 infection assay by treating the edited CD4^+^ T cells with HIV-1_NL4-3_ and HIV-1_YU-2_ (1:1) simultaneously. Interestingly, the results showed that, compared with single modified group (CCR5, CXCR4-1, CXCR4-2) or unmodified control, the simultaneous co-receptor edited group (#1,#2) showed a significant dual-tropic HIV-1 variant protection at different time points (Fig. 5d).

### CXCR4 and CCR5-modified primary CD4^+^ T cells do not have a significant off-target effect

**Fig. 6:**
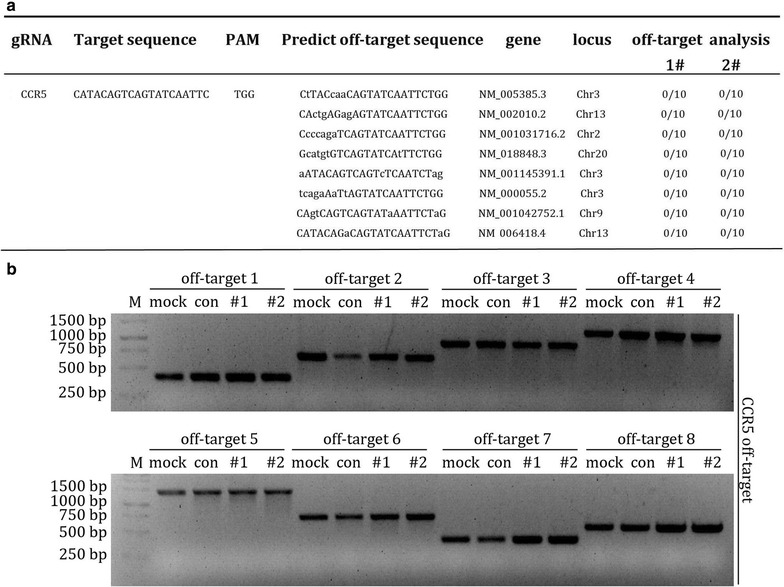
off-target analysis of CCR5. **a** Mutation frequency analysis at predicted off-target sites of CCR5. The off-target sites were predicted and aligned with the human genome. The sites were amplified and cloned into T-vector and subjected to sequencing. **b** T7E1 analysis of all predicted off-target sites

To test the specificity of the X4R5-Cas9 system in disrupting CXCR4 and CCR5, the predicted sgRNA off-target sites were analyzed. For predicted off-target sites of CXCR4 (sgX4-1,sgX4-2), our previous results showed that no off-target effects could be identified by DNA sequencing of each fragments [37]. Meanwhile, we further performed deep sequencing or T7E1 of all the predicted off-target sites in this study, and the data support our previous conclusion (Additional file 2: Figure S1). For the predicted off-target sites of CCR5 (sgR5), DNA sequencing and T7E1 results showed no obvious indels at the eight predicted off-target sites in the genome of lenti-X4R5-Cas9 edited cells (Fig. 6a, b).

### No difference of apoptosis observed in lenti-X4R5-Cas9 modified CD4^+^ T cells

**Fig. 7:**
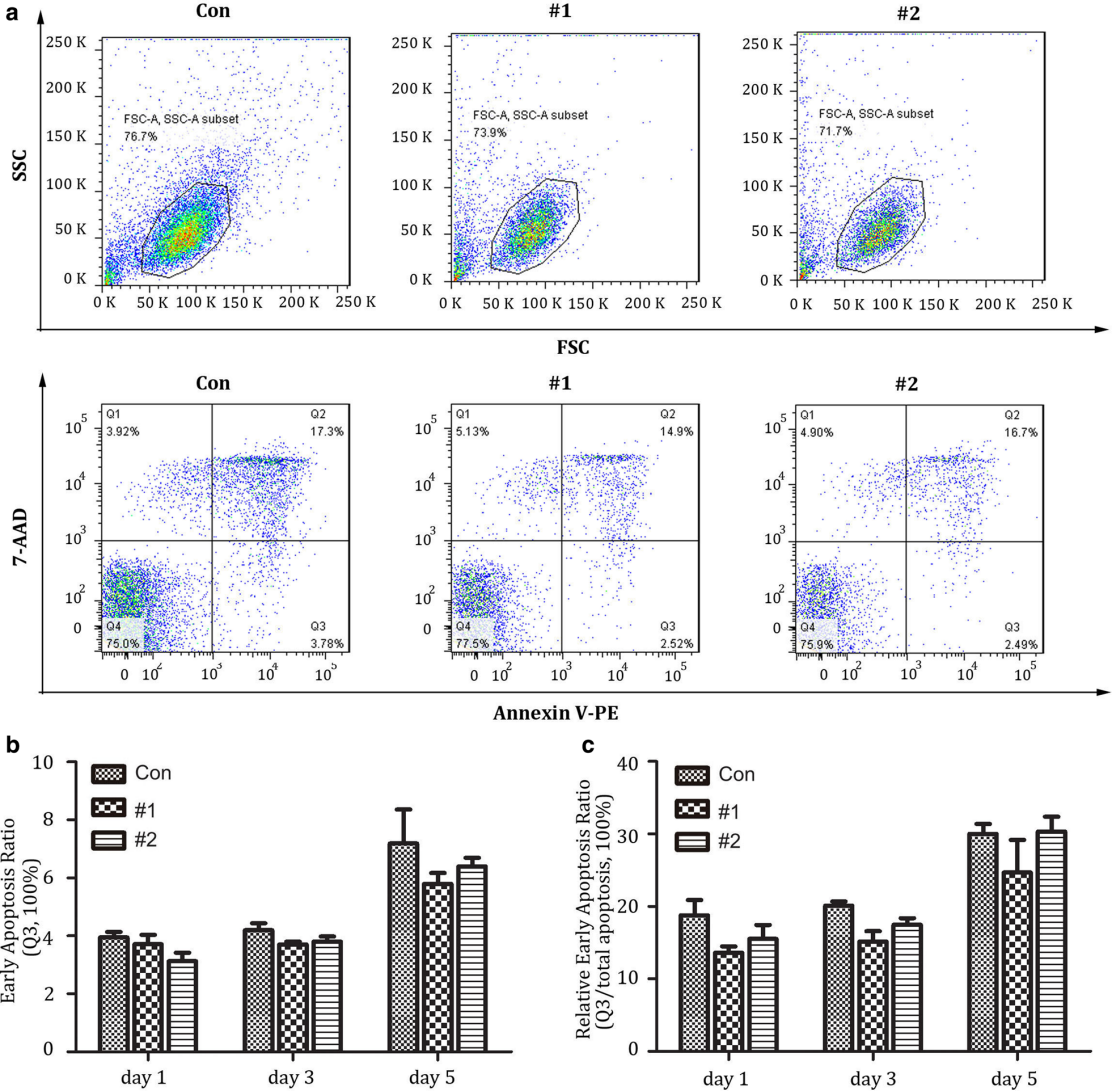
Apoptosis analysis after lenti-X4R5-Cas9 modification in primary CD4^+^ T cells. **a** Annexin V and 7-AAD were utilized to stain modified CD4^+^T at 1,3 and 5 days post nucleofection with flow cytometry. Necrotic cells (Annexin V-/7AAD +), necrotic or late apoptotic cells (Annexin V +/7AAD +); early apoptotic cells (Annexin V +/7AAD-); viable cells (Annexin V-/7AAD-). **b** Early apoptosis ratio at day 1,3,5 post gene disruption. **c** relative early apoptosis ratio (Q3/total none viable cells). The total none viable cells = Q2 + Q3. The data shown were the mean ± SD of three independent experiments. *P < 0.05

To test whether the lenti-X4R5-Cas9 mediated disruption of CXCR4 and CCR5 on CD4^+^ T cells causes cellular toxicity or apoptosis, the modified cells were analyzed for early apoptosis using flow cytometry assay with 7-aad and Annexin V. The results indicated that, compared with unmodified control cells, no significant difference in apoptosis was observed at day 1 to day 5 post-transfection in X4R5-gene edited cells (Fig. 7a, b, c).

## Discussion

In this study, we found that CRISPR/CAS9 could efficiently disrupt the HIV co-receptors CXCR4 and CCR5 simultaneously using combined sgRNAs targeting both *CXCR4* and *CCR5* gene sequences. The high on-target ratio in TZM-bl, Jurkat T cells, and human CD4^+^ T cells were shown to protect cells from X4- or/and R5-tropic HIV-1 infection. The CRISPR/Cas9 system was demonstrated to be tolerable of mismatch between designed sgRNAs and its targets [42]. In our study, off-target analysis was performed, and no evident off-target was detected in any of the predicted sites in primary CD4^+^ T cells, which confirmed the specificity of our designed sgRNAs. To exclude potential toxicity after the disruption of CXCR4 and CCR5 in primary CD4^+^ T cells, the apoptosis ratio analysis revealed that CXCR4 and CCR5 disruption did not cause the cells to experience cytotoxicity as compared with unmodified cells, which indicated that the disruption was relatively safe for gene therapy in the future.

From previous studies, HIV-1 enters cells via co-receptor CCR5 at the early stage of infection and many studies involving CCR5 disruption have been performed by utilizing gene manipulation tools, such as ZFN, TALEN, and the newly developed CRISPR/Cas9 [22, 33, 43, 44]. Meanwhile, CXCR4, which may act as a co-receptor for late stage entry and accelerate the progress of the disease [34], has also been studied [35, 37]. In 2015, Poveda et al. reported that CCR5 disruption might drive the HIV-1 co-receptor switch, which caused HIV-1 entry cell through CXCR4 [36]. From a clinical study, the *Essen Patient*, who was a 27-year-old patient with HIV-1 infection and anaplastic large-cell lymphoma, received a HLA-compatible CCR5∆32 stem cell transplantation, which was similar to the *Berlin Patient.* However, the outcome was that the virus switched to be CXCR4 preferential X4-tropic HIV-1, which indicated that editing only CXCR4 or CCR5 may not be sufficient [45]. Therefore, simultaneous HIV co-receptor disruption should be considered. Although studies about CXCR4 and CCR5 editing simultaneously in cells are limited. In 2014, Chuka Didigu et al. reported that they had successfully edited CXCR4 and CCR5 simultaneously by ZFN in various cells including Sup T1-R5 and primary CD4^+^ T cells [21]. Excitingly, their study of the NSG mouse transferred with modified primary CD4^+^ T cells showed a significant survival advantage in the presence of HIV-1 infection in vivo compared to the unmodified group. Yu et al. also reported recently that they have successfully disrupted CXCR4 and CCR5 simultaneously by CRISPR/Cas9 in cell lines and primary CD4^+^ T cells [38]. Compared with complicated ZFN approaches, the CRISPR/Cas9 technology provides appropriate target sites and may be simpler in design and vector construction. However, for specificity and efficiency, more CXCR4 and CCR5 target sites should be considered in the study of simultaneous editing of the two genes by CRISPR/Cas9. In our study, we further confirmed that simultaneously editing of CXCR4 and CCR5 by CRISPR/Cas9 with dual-sgRNAs in single vector could function in various cell lines. In addition, compared with Yu et al. study, we designed and studied another two different sgRNA combinations (two sgRNAs for *CXCR4*, one sgRNA for *CCR5*), and the cleavage efficacy from T7E1, DNA sequencing, or flow cytometry showed that both sgRNA combinations could efficiently disrupt the specific genes. The selection of sgRNAs for *CXCR4* were screened from 10 designed candidates from our previous report [37]. Moreover, the updated T7E1 and deep sequencing analysis of the predicted off-target sites of sgX4-1 and sgX4-2 in this study have also verified the specificity (Additional file 2: Figure S1). Meanwhile, the selected sgRNA of CCR5 (sgR5) targets the natural ∆32 region, which leads to an artificial mutation similar to the natural ∆32 mutation, and introduces a premature stop code. The CCR5 mutant will generate a truncated protein that is not expressed on the cell surface. Importantly, the sgR5 we selected for combination was also used by Ye et al. to generate induced pluripotent stem cells (iPSCs) homozygous for the naturally occurring CCR5∆32 mutation with PiggyBac technology [39]. Lots of previous work or clinical trails showed that disruption of the ∆32 domain can confer significant HIV resistance in the edited cells and the domain may be considered as the most potential gene editing target [15, 18, 45–47]. DNA sequencing or T7E1 analysis also indicated that the sgR5 in the two X4R5-Cas9 plasmids also had a high on-target efficacy and without obvious off-target effects. Furthermore, compared with Yu et al. work, we have also tested Jurkat T cells, and the disruption efficacy of packaged X4R5-Cas9 lentivirus in this cell was also confirmed. The selective advantage assay in modified cells over unmodified cells was also performed in Jurkat cells. Interestingly and importantly, both studies verified that simultaneous modification of CXCR4 and CCR5 rendered the cells more resistant to dual-tropic HIV-1 variant, compared to none or single disruption of CXCR4 or CCR5 in primary CD4^+^ T cells. Thus, to some extent, both studies have verified the efficacy and potential of CRISPR/Cas9 in simultaneous HIV-1 co-receptor editing with different sgRNA combinations.

It should be noted that CXCR4 has been identified to have a critical role in sustaining normal physical function of hematopoietic stem cells [48, 49]. However, the disruption of CXCR4 in T cells in mice did not show humoral or cell response differences compared to untreated mice, which suggests loss of CXCR4 in T cells may be immune tolerant [50]. Meanwhile, ZFN mediated CXCR4 disruption also presented no evident effect on cell proliferation ability [21, 35]. In addition, our previous study about CXCR4 editing by CRISPR-Cas9 in CD4^+^ T cell also showed the same result [37]. In this study, we also noticed that modification of both CXCR4 and CCR5 showed no obvious influence on cellular proliferation. About the disruption efficacy of the two constructed plasmids, interestingly, we have noticed during the study that the lenti-X4R5-Cas9-#1 may work better than lenti-X4R5-Cas9-#2 even with the same CCR5 sgRNA sequence in CCR5-tropic HIV-1_YU-2_ challenge assay, especially in TZM-bl cells and Jurkat T cells. The reason may be due to different expression of CCR5 sgRNA driven by U6 promoter or synergetic CXCR4 gene disruption effect of different sgRNAs. Furthermore, it should also be noted that the editing efficacy and resistance to HIV-1 infection could be further verified if we could transfer the system into the NSG immunodeficient mice for in vivo studies. Besides, from presented T7 endonuclease 1 and DNA sequencing analysis of lenti-X4R5-Cas9 mediated modification of CXCR4 and CCR5 in each cell type we selected, it shall be that the plasmid delivery efficiency in TZM-bl and Jurkat T cells were positive and much more high than in primary CD4^+^ T cells and any other effective and safe delivery methods should be further investigated and applied for primary CD4^+^ T study.

## Conclusions

In this study, we designed two different combinations of sgRNAs targeting CXCR4 and CCR5 simultaneously by CRISPR/Cas9 system, which showed high specificity and no evident toxicity on cells after gene disruption. We demonstrated that the genome-edited cells could efficiently protect cells from X4- or/and R5-tropic HIV-1 infection and the modified cells take selective advantage over unmodified cells during the HIV-1 infection. Our results suggest that dual-modification of CXCR4 and CCR5 by CRISPR/Cas9 can represent an effective and safe approach in HIV gene therapy and may have potential clinical application.

## Additional files

**Additional file 1: Table S1.** Primers used in this study. a, primers used for CXCR4,CCR5 fragments as well as vector construction. PX4K represents PacI-U6-sgX4-1/-2-KpnI. b, primers for each predicted off-target site of CCR5 study. c, primers used for amplification of CXCR4 off-target sites.

**Additional file 2: Figure S1.** Off-target analysis of CXCR4 by deep sequencing and T7E1. The fragments of predicated off-target sites were amplified and purified. The amplicons were then sent for deep sequencing. a, off-target sites of CXCR4 (sgX4-1) from lenti-X4R5-Cas9-#1. b, off-target sites of CXCR4 (sgX4-2) from lenti-X4R5-Cas9-#2. c, T7E1 analysis of all predicted off-target sites.
