# Supplementary figures and images for: Genome editing of the HIV co-receptors CCR5 and CXCR4 by CRISPR-Cas9 protects CD4+ T cells from HIV-1 infection
Source: Cell Biosci. 2017 Sep 9;7:47. doi: 10.1186/s13578-017-0174-2 (PMC5591563; doi:10.1186/s13578-017-0174-2)

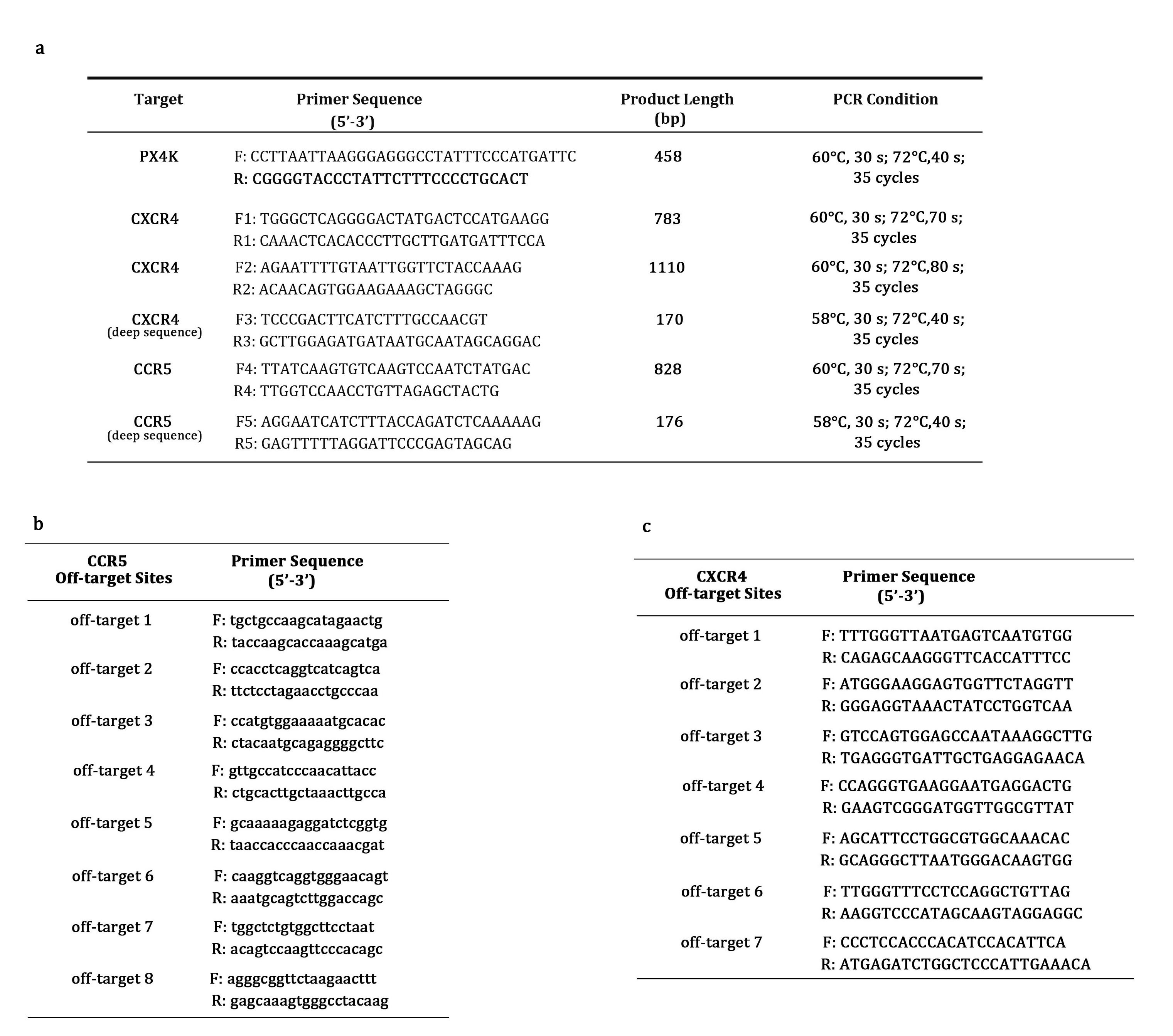

Supplement: Supplementary file 1 — Additional file 1: Table S1. Primers used in this study. a, primers used for CXCR4,CCR5 fragments as well as vector construction. PX4K represents PacI-U6-sgX4-1/-2-KpnI. b, primers for each predicted off-target site of CCR5 study. c, primers used for amplification of CXCR4 off-target sites. [file 13578_2017_174_MOESM1_ESM.jpg]

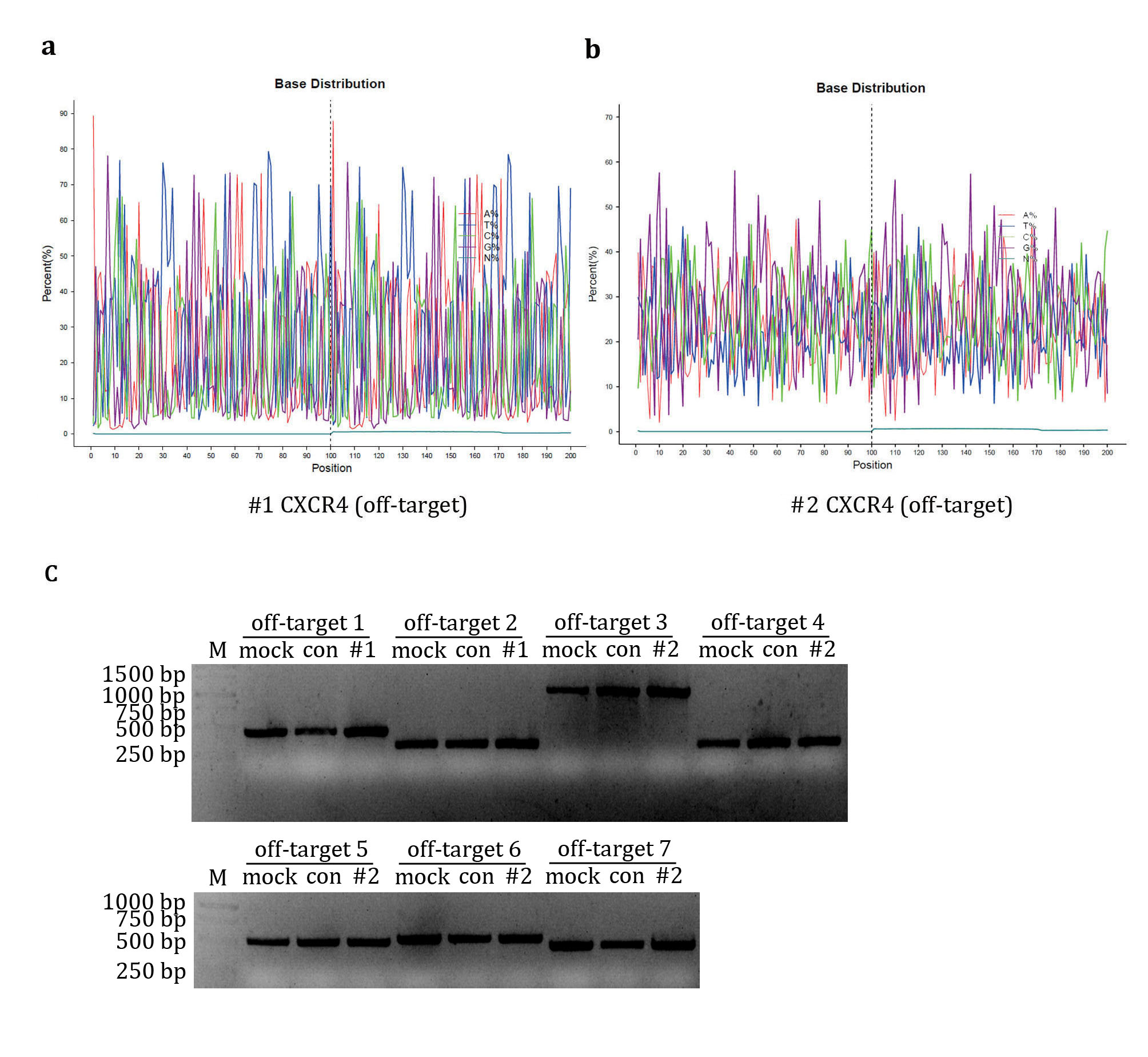

Supplement: Supplementary file 2 — Additional file 2: Figure S1. Off-target analysis of CXCR4 by deep sequencing and T7E1. The fragments of predicated off-target sites were amplified and purified. The amplicons were then sent for deep sequencing. a, off-target sites of CXCR4 (sgX4-1) from lenti-X4R5-Cas9-#1. b, off-target sites of CXCR4 (sgX4-2) from lenti-X4R5-Cas9-#2. c, T7E1 analysis of all predicted off-target sites. [file 13578_2017_174_MOESM2_ESM.jpg]
